# Food taboos and myths in South Eastern Nigeria: The belief and practice of mothers in the region

**DOI:** 10.1186/s13002-016-0079-x

**Published:** 2016-01-27

**Authors:** Uchenna Ekwochi, Chidiebere D. I. Osuorah, Ikenna K. Ndu, Christian Ifediora, Isaac Nwabueze Asinobi, Christopher Bismark Eke

**Affiliations:** Department of Paediatrics, Enugu State University of Science and Technology, Enugu State Enugu, Nigeria; Child Survival Unit, Medical Research Council UK, The Gambia Unit, Fajara, The Gambia; Griffiths University Medical School, Gold Coast, Australia; Department of Paediatrics, College of Medicine, University of Nigeria, Enugu State Nsukka, Nigeria

**Keywords:** Food taboos, Pregnancy, Under-two years, Enugu

## Abstract

**Background:**

Poor nutritional practices especially in pregnancy and early childhood can result in dire consequences in the growth and development of a child.

**Methods:**

This study using purposive sampling enrolled 149 women who had carried at least one pregnancy to term in Enugu south east Nigeria. Logistic regression analysis was used to assess association between avoidance of certain food in pregnancy and selected socio-demographic factors.

**Results:**

Approximately 37 % of respondents avoided some foods in pregnancy due to food taboos and no relationship was seen between this avoidance of food and maternal educational attainment, parity (number of obstetrics deliveries) and occupation. Snail and grass-cutter meat were the commonly avoided food in pregnancy while egg were commonly avoided in children under-two years old. Some respondent believed eating snail and grass-cutter meat makes a child sluggish and labour difficult respectively while starting egg early for a child could predispose them to stealing later in life.

**Conclusion:**

Discussion about food taboos during antenatal care visits and during community education can help reduce the traditional belief about certain food in pregnancy and early childhood.

## Background

James D’Adamo authored a book titled “One Man’s Food…is Someone Else’s Poison” [1]. This implies that what appeals to a particular group of people as delicacies may actually be unappealing and in extreme cases forbidden to another group of people. The word taboo in general terms is a belief that forbids association of a group people with other people, places or practices [2]. Food taboos which is a type of these taboos, represents unwritten social rules mainly based on religious and/or historical reasons that regulate food consumption in a community [3]. Barfield stated that there may be as many as 300 reasons for particular avoidance [4].

According to the UNICEF Food-Care Health conceptual framework, cultural norms, taboos and beliefs lie within the contextual factors included as one of the basic causes of malnutrition [5, 6]. Food taboos which is a relatively commoner among poor communities especially in Sub-Saharan Africa is often more strictly practiced by pregnant and lactating women to prevent what they perceive as harmful effect of these foods on the newborn [7, 8]. This practice was described in the Gambia where due to some traditional belief, women of ‘Fulla’ ethnicity are usually forbidden from eating several types of food rich in carbohydrate, animal proteins, and micronutrients during pregnancy [9]. The study hypothesized that the food taboos maybe a contributing factor to the high protein-caloric malnutrition during childhood and pregnancy among this ethnic group [9]. Inadequate intake has been shown to be a major contributor to malnutrition which is an underlying factor in more than 50 % of the core causes of childhood deaths in developing countries [10–12]. Other studies have associated certain maternal socio-demographic factors with adherence to food taboos [13, 14].

In line with this fact, the UNICEF advocated improving and expanding delivery of key nutrition interventions during the critical 1000-day window covering a woman’s pregnancy and the first two years of her child’s life, when rapid physical and mental development occurs [15]. In sum, cultural norms, taboos and beliefs may influence what mothers eat during pregnancy and this in turn may affects the birth weight and subsequent wellbeing of their babies. Such taboos may also influence what mother feed their children. This study was designed to identify the various food taboos during pregnancy and in under-two children in the South Eastern Nigeria and the reasons for avoiding these foods. It is believed that identifying the food taboos practiced by the participants in this study will help entrench Cultural Competency in the delivery of child and maternal health care services in South Eastern Nigeria.

## Methods

### Description of study area

This is a hospital based study conducted over a 3 months period (November 2014- January 2015) in the Antenatal clinics of Enugu State University Teaching Hospital (ESUTH) in Parklane Enugu, south eastern part of Nigeria. ESUTH is a tertiary health facility located in the state’s metropolis and serves as a referral center to other secondary and primary health facilities within the state and its environs. Enugu state has a population of 2,125,068 people as at 2005, with an average annual g rowth rate of 3 % and literacy rate of 66 % [16]. Majority of its residents are of Igbo ethnicity and Christianity is the dominant religion.

### Enrolment of respondents

This is a cross-sectional with purposive sampling method employed in enrolling study participants. Pregnant mothers presenting to the clinics and those who consented to participate were consecutively enrolled. They were interviewed on their belief and practice of food taboos during pregnancy and in their young children. The interview was done using a structured pretested questionnaire. Further interviews were conducted on individuals who admitted to avoiding certain food to ascertain their reasons for such practice. All interviews were carried out by medical students in their final years that were trained for two weeks on how to administer the questionnaires. Daily quality checks were also done to detected errors and correction initiated.

### Respondent’s socio-demographic characteristics

The age, parity (number of obstetrics deliveries), ethnicity, religion, highest education attainment and occupation of respondents were ascertained. Age was categorized into *<20, 21–25, 26–30, 31–35, 36–40 and >40 years*. Parity was grouped into *1st, 2nd to 5th and >5th pregnancy*. Ethnicity was grouped into *Igbos, Hausas, Yorubas* and others. Religion of the respondents was categorized as *Christianity, Muslim, and Traditional religions*. The highest education level and occupation of the respondents were assessed using Olusanya and Okpere socio-economic classification [17]. The highest education level was grouped as; *(i) University graduate or equivalent, (ii) O’level or Senior School certificate (SSCE) who also had teaching or other professional training (iii) school certificate or grade 11 teachers certificate or equivalent (iv) Juniour Secondary School Certificate (JSSCE), primary six certificate (v) no education*. The occupation of respondents were stratified into*; (i) Skilled occupation* i.e. senior public servants, professionals, managers, large scale business men, contractors *(ii) Semi-skilled occupations* i.e. intermediate grade public servants, senior school teachers, *(iii) Unskilled occupation* i.e. junior school teachers, drivers, artisans*,* petty traders, laborers, messengers *(v) unemployed* i.e. full time house wife, student, subsistence farmer etc.

### Belief and practice of food taboos among respondents

Respondents were interviewed to ascertain whether or not they believe traditional taboos that certain food should not be eaten by pregnant women or children under two years of age. They were asked to state whether or not they practice such beliefs and to list the various food items that are associated with taboos in their locale. Among respondents who practice food taboos, a few were randomly selected for a more detailed interaction to ascertain the various reasons for avoiding such food in pregnancy and in their younger children.

### Data management

Analysis was done with SPSS Version 22. Results were presented in tables and charts. Apart from frequencies, significant associations between the food taboos practice and a few independent respondent-variables were also explored using Binary Logistics Regression. To facilitate analysis in this regard, the responses to Parity, Educational Attainment and Occupation were dichotomized, and these represent the independent variables. The responses to the practise of food taboos categorized as Yes (for those that practised) and No (for those that do not practise) was the dependent variable. Results of the Regression Analysis was presented as Odd Ratios (ORs) with 95 % Confidence Intervals provided and significance level at <0.05.

## Ethical consideration

Ethical clearance was obtained from the Enugu State University Teaching Hospital Ethics Committee. Informed consent was obtained from every mother in her own right before recruitment. Participation in the study was entirely voluntary and no financial inducement whatsoever was involved. All information was handled with strict confidentiality.

## Results

Table 1 summarizes the demographics of the respondents. There were 149 responses out of 200 questionnaires distributed, giving a response rate of 74.5 %. Out of these 149 respondents, majority (39.6 %) were within the 26–30 age brackets and slightly over a quarter (25.5 %) was 25 years or less. Nearly two-thirds (64.8 %) of the respondents have had 2–5 pregnancies while 39 (26.9 %) and 12 (8.3 %) have had only one and more than five pregnancies respectively (Table 1). The vast majority of respondents are Christians (97.3 %) of Igbo ethnic group (96.0 %). Seventy-six (51.0 %) of the surveyed women were educated to the university level or equivalent, while 7 (4.7 %) are not educated at all. Finally, most of the respondents had skilled (28.5 %) and unskilled (27.8 %) occupations while the remaining were semi-skilled workers (17.4 %) or unemployed (26.4 %). One hundred and thirty-seven out of the 149 responses answered questions relating to practice of food taboos. Fifty (36.5 %) admitted avoiding certain foods in pregnancy and in their young children based on believe of food taboos while 85 (63.5 %) did not.

**Table 1:** Basic response characteristics of the respondents

Socio-demographic factors	Number (N)	Percentages (%)
Age (years)	*N* = 149	
≤25	38	25.5
26–30	59	39.6
31–35	34	22.8
≥36	18	12.1
Parity	*N* = 145	
1st pregnancy	39	26.9
2nd–5th pregnancy	94	64.8
>5th pregnancy	12	8.3
Ethnicity	*N* = 149	
Igbo	143	96.0
Other ethnic groups^a^	6	4.0
Religion	*N* = 149	
Christianity	145	97.3
Others^b^	4	2.7
Highest education	*N* = 149	
University or equivalent	76	51.0
Secondary ± other professional training	50	33.6
Primary Education	16	10.7
No education	7	4.7
Occupation	*N* = 144	
Skilled	41	28.5
Semi-skilled	25	17.4
Unskilled	40	27.8
Unemployed	38	26.4

^a^other ethnicity included Hausa and Yoruba; ^b^other religion included Islam and traditional practice

## Reasons for avoiding certain foods in pregnancy based on taboos

**Fig. 1:**
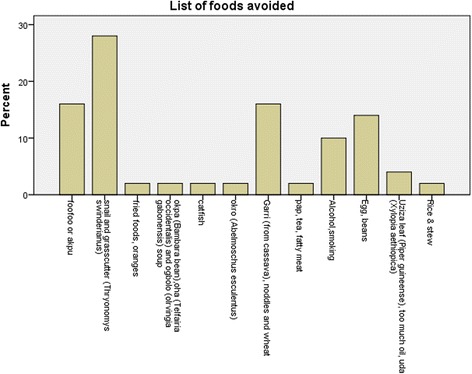
Show the major groups of food avoided in pregnancy on taboo grounds

Figure 1 shows the list of food respondents avoided during pregnancy. When interviewed further on the reason why she avoided some of the listed food items, a 32 year old woman (AJ) in the second trimester of her fourth pregnancy had this to say;

{AJ, 32 years, gravida 4}; *“I cannot eat snail in pregnancy because it will make my baby to be sluggish in life and spit too much saliva.”*

A 28 year old woman in the last trimester of her second pregnancy stated that;

{CM, 28 years, gravida 2}; *“I will not eat bush meat like Grasscutter (Thryonomys swinderianus) when I am pregnant because it will cause my labour to be difficult and prolonged during delivery”.*

Another older woman who has had six babies narrated thus;

{ABJ, 34 years, gravida 6}; “*I avoid starchy food like ‘garri’ (cassava flakes) and nodules in pregnancy because they will make my baby to have excess weight which will be difficult to deliver except by operation”*

**Fig. 2:**
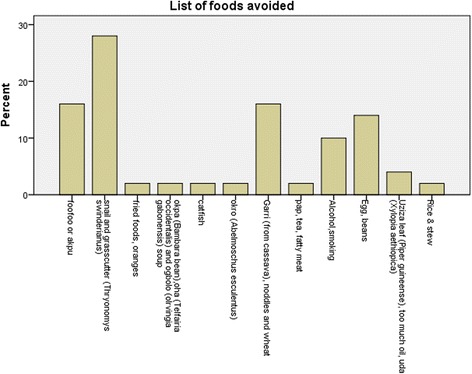
Foods avoided in under-2 children based on taboos

Figures 2 summarized the foods generally avoided in children aged two years or below by the respondents. The various reasons respondents avoided the above food items in children less than two years as stated included;

{ZE, 28 years, multigravida}; *I don’t give young children egg because it will cause them to start stealing because it is very sweet”.*

{MA, 33 years, gravida 2}; *“I don’t allow my children to ‘drink garri’ because it causes eye problem’*

{UC, 29 years, multigravida}; *“I avoid ‘sweet’ in small children because it causes worm”*

Table 2 summarizes the findings on logistic regression that explored the associations between the avoidance or otherwise of foods in pregnancy based on taboos and a few respondent variables. The three respondent variables and their groups include parity, occupation and highest educational qualifications. As shown on the table, none of these variables were significantly associated with avoidance of certain foods due to food taboos in pregnancy.

**Table 2:** Binary logistic regression on food avoidance based on taboos among respondents

Variable (Definition)	Estimate	Odds Ratio	OR (95 % CI)		
			Lower	Upper	*P*
Age (≤30 years vs. > 30 years)	- 0.5	0.61	0.27	1.39	0.24
Parity (Multigravida vs. Primigravida)	0.78	2.20	0.89	5.45	0.09
Highest educational Attainment (University/equivalent vs. Non-university degree/equivalent)	0.40	1.49	0.67	3.28	0.38
Occupation (High-profile careers vs. Low-profile careers)	- 0.37	0.69	0.31	1.55	0.37

## Discussion

Approximately half of the respondents in this study admitted avoiding one food or the other in pregnancy and in their young children based on the associated food taboos. Similarly, Bartholomew and Poston found that 50 % of women had traditional food beliefs and as much as 10 % rejected nutritious foods which interfered with adequate prenatal nutrition [18]. The finding that slightly above half of the respondents in our study do not adhere to these taboos is similar to the report amongst the Lese women of the Ituri forest in northeast Democratic Republic of Congo. These women were found to cope with these restrictions by either secretly ignoring them or by eating nutritious foods that supposedly prevented the consequences of eating tabooed foods [17]. It was inferred by the authors of the study that most women who avoided these foods were coerced into such practise and if given the choice will eat such foods in pregnancy [19].

Our study revealed that the foods most commonly avoided in pregnancy were Snail and Grass-cutter meat. Snails are avoided because it is believed that they make babies sluggish and salivate excessively like a snail. Prolonged labor was the major reason given for avoiding grass-cutter meat. Maduforo [20] also found the same traditional belief in a study about the traditional and nutritional habits among pregnant women in a rural Nigerian setting. Many cultures portray eating snail meat as taboo and believe that it makes an individual sluggish or slow and link this to the slimy nature of the snail’s secretions [21]. Islam and the Jewish faith also prohibit eating of snail meat [22]. In Yilo Krobo District in Ghana, snail is also among the list of foods prohibited in pregnancy. In this district, in addition to concern for healthy pregnancy and outcome, respect for ancestors were the common reason for such prohibition [23].

There has not been any established link between snail consumption and sluggishness and grass cutter consumption with prolonged labour. On the contrary, the giant African snail (Archachatina marginata) has been shown to be a rich source of protein, trace elements and minerals which are needed for proper growth and development in human beings [24]. In the same vein, the grass cutter or cane rat (Thryonomys spp) is also a known source of rich animal protein [25]. These foods are cheap and can serve as commonly available sources vital nutrients for a balanced diet in developing countries. Their consumption could therefore reduce maternal malnutrition if utilised fully.

It was also found in this study that the food most commonly denied children is egg which is a source of high biological value protein. The belief is that it leads children to theft. This practice has been reported by other studies [20, 26] and the relationship between eggs and theft has no basis in science. The current study further revealed that parity, educational level or occupation had no significant relationship with avoidance of foods due to associated taboos. Thus maternal experience or socioeconomic status may not necessarily guarantee nutrition knowledge or the application of this knowledge for practical purposes and this demonstrates the effect that traditional beliefs and cultural norm can have on human behaviour in general. Policy makers and health care providers may look at the findings from this work as they formulate strategies that will be more culturally-sensitive to the the dietary needs of the patients involved, while upholding best clinical practice. As established in existing literature [27–29] boosting cultural competence in this regard will help improve health outcomes and quality.

## Conclusion

Food taboos still contribute to unhealthy nutritional practices in pregnancy and early childhood. These findings therefore underscore the need to address food myths and taboos during antenatal visits and in major nutritional campaigns targeted at pregnant women and communities with traditional believe about certain food.
